# Evaluation of Conventional Polymerase Chain Reaction for Accurate Species Identification of Elizabethkingia Compared to Matrix-Assisted Laser Desorption/Ionization Time-of-Flight Mass Spectrometry

**DOI:** 10.7759/cureus.87087

**Published:** 2025-07-01

**Authors:** Sailaxmi Mahapatra, Ashoka Mahapatra, Sushree Sarathi, Bijayini Behera, Sarita Mohapatra

**Affiliations:** 1 Microbiology, All India Institute of Medical Sciences, Bhubaneswar, Bhubaneswar, IND; 2 Microbiology, All India Institute of Medical Sciences, Raipur, Raipur, IND; 3 Microbiology, All India Institute of Medical Sciences, New Delhi, New Delhi, IND

**Keywords:** conventional pcr, e. anophelis, e. meningoseptica, maldi-tof ms, vitek 2

## Abstract

Background: Accurate identification of *Elizabethkingia* species is crucial for clinico-microbiological analysis and enabling appropriate antibiotic therapy. *Elizabethkingia anophelis* (*E. anophelis*) is frequently misidentified as *Elizabethkingia meningoseptica* (*E. meningoseptica*) by VITEK 2 (bioMérieux SA, Marcy‑l’Étoile, France), while matrix-assisted laser desorption/ionization time-of-flight mass spectrometry (MALDI-TOF MS) provides correct identification. However, MALDI-TOF MS is not widely available. Polymerase chain reaction (PCR) amplification targeting specific genes has shown promising accuracy.

Objective: The objective of this study was to perform genotypic identification of *Elizabethkingia *isolates to the species level using conventional PCR and compare the results with MALDI-TOF MS as the gold standard.

Materials and methods: Archived *E. meningoseptica *isolates from bloodstream infections (BSI) were subjected to DNA extraction using HiMedia Kits (HiMedia Laboratories Pvt. Ltd., Mumbai, India). Conventional PCR was performed with specific primers for *E. anophelis *and *E. meningoseptica*. Amplicons were analyzed by gel electrophoresis and visualized using an ultraviolet (UV) transilluminator (Syngene, Germany).

Results: PCR results were compared to those of MALDI-TOF MS. All isolates were identified as *E. anophelis *with 100% sensitivity and specificity. No bands matched *E. meningoseptica*.

Conclusion: Conventional PCR with specific primers for *E. anophelis *and *E. meningoseptica *is a reliable and cost-effective alternative to MALDI-TOF MS for accurate species-level identification of *Elizabethkingia*.

## Introduction

*Elizabethkingia*, a hospital-acquired bacterium, has become a major clinical concern, especially in intensive care units (ICUs), due to its impact on patient management and outcomes [1]. Within this genus, the principal species responsible for human infections are *E. meningoseptica* and *E. anophelis *[2].

*E. meningoseptica *is a well-known pathogen in neonatal care, particularly associated with meningitis in premature infants during the early weeks of life. It also serves as an opportunistic organism in various other infections [1]. In contrast, *E. anophelis*, a recently discovered species identified in 2011, has been associated with sporadic outbreaks in regions such as the United States, Hong Kong, and Taiwan [3]. A notable series of *E. anophelis *infections was reported by the Centers for Disease Control and Prevention (CDC) in 2016 across Wisconsin, Michigan, and Illinois, which were determined to be independent cases rather than a single-source outbreak [4].

Advancements in identification techniques, particularly MALDI-TOF MS, have revealed that many isolates formerly classified as *E. meningoseptica *were in fact *E. anophelis *[5]. Supporting this, clinical studies in Singapore found *E. anophelis *to be the most prevalent *Elizabethkingia *species in bloodstream infections [6].

The treatment of *Elizabethkingia *infections is complicated by intrinsic resistance to multiple antibiotic classes, particularly β-lactams and aminoglycosides. Comparatively, *E. meningoseptica *often presents with higher minimum inhibitory concentrations (MICs) for several antibiotics, making it more resistant and commonly associated with severe diseases such as meningitis and sepsis [7].

Traditional biochemical identification methods fall short in differentiating between *Elizabethkingia *species. These bacteria typically form oxidase-positive, translucent, non-lactose fermenting colonies that yield K/K reactions on triple sugar iron (TSI) agar and are non-fermenters in oxidative-fermentative testing. Notably, they resist colistin while remaining sensitive to vancomycin [8]. Although the VITEK 2 system (bioMérieux SA, Marcy‑l’Étoile, France) is widely used, it often misidentifies *E. anophelis *as *E. meningoseptica*, whereas MALDI-TOF MS provides more accurate results [9].

A study conducted at AIIMS, Bhubaneswar, reported that all 80 isolates initially identified as *E. meningoseptica *using VITEK 2 were later confirmed to be *E. anophelis* by MALDI-TOF MS (bioMérieux SA, Marcy‑l’Étoile, France) at All India Institute of Medical Sciences (AIIMS), New Delhi [8]. Given the limited availability of MALDI-TOF MS in routine settings, molecular tools like PCR targeting specific gene sequences could provide effective alternatives.

Chew et al. demonstrated the utility of PCR in distinguishing *E. anophelis *and *E. meningoseptica*, using primers targeting the genes encoding lipid A-disaccharide synthase and a sodium-proton antiporter, respectively. Their method showed high specificity without cross-reactivity, underlining the clinical relevance of accurate species identification for guiding antibiotic therapy [6].

Considering the scarcity of Indian studies and the frequent misidentification observed in earlier findings, our study aims to apply conventional PCR for genotypic identification of *Elizabethkingia *species and compare the results to MALDI-TOF MS, the current reference standard.

## Materials and methods

A total of 80 archived *Elizabethkingia* spp. isolates from bloodstream infection (BSI), stored at −80 °C in glycerol slants, were revived using brain-heart infusion (BHI) broth. These were subcultured onto blood and MacConkey agar, yielding translucent, non-lactose fermenting (NLF) colonies. Genomic DNA was extracted using the spin column method (HiPurA® Bacterial Genomic DNA Purification Kit, MB506-20 PR, Mumbai, India), and DNA purity was evaluated via 260 nm absorbance measurements. The purified DNA was stored at −20 °C for subsequent analysis.

PCR was conducted for all the isolates using DreamTaq Green PCR Master Mix (Thermo Fisher Scientific Inc., Waltham, Massachusetts) in a 25 μL reaction containing 25 ng of template DNA. The primer sets used for *E. anophelis* were anoR (5'-TGCGTTATTACCAGGTAGTCGG-3') and anoF (5'-GACTTCCGCGGTAGCAAACAA-3'), while mengF (5'-TGGGACCTATTGCTGTTGGTT-3') and mengR (5'-ACCACTTCCTGTGTACCTGC-3') were used for *E. meningoseptica*. *E. coli *ATCC 25922 was used as the negative control. MALDI-TOF identified *E. anophelis* as the positive control.

The thermal cycling protocol involved an initial denaturation at 95 °C for 5 minutes, followed by 35 cycles of denaturation (94 °C for 30 seconds), annealing (55 °C for 30 seconds), and extension (72 °C for 30 seconds), with a final extension at 72 °C for 10 minutes. Amplicons (5 μL), along with a 100 bp DNA ladder, were electrophoresed on 1.5% agarose gels and visualized under a UV transilluminator (Syngene, Germany).

## Results

**Figure 1:**
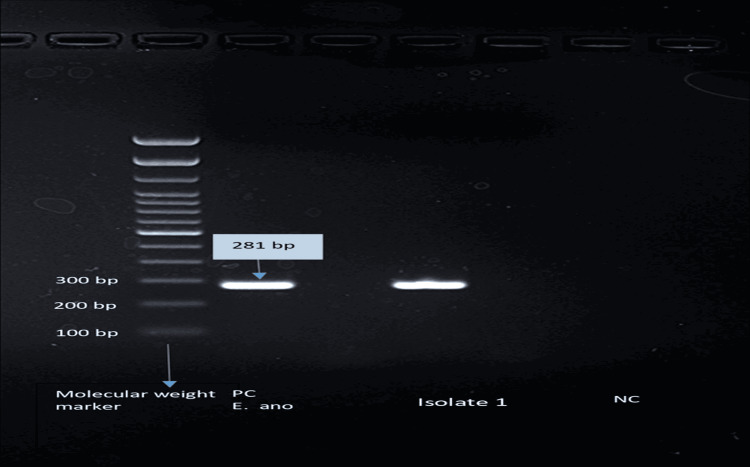
Gel electrophoresis photograph of conventional PCR using Elizabethkingia species-specific primers. Each isolate was subjected to primers for *E. anophelis *(anoF and anoR) and *E. meningoseptica *(mengF and mengR). A band size of 281 bp indicates a positive result for *E. anophelis*. Isolate 1 shows a positive band (281 bp) for *E. anophelis *and negative for *E. meningoseptica*. NC: negative control; PC: positive control; PCR: polymerase chain reaction.

During the study period (August 2020 to December 2021), a total of 13,747 blood culture samples were processed. Of these, 1,869 samples (13.59%) were found to be culture-positive. Among the positive cultures, 273 isolates (14.60%) were identified as non-fermentative Gram-negative bacilli (NFGNB). Within this group, *Elizabethkingia *species accounted for 29.30% (80/273) of the isolates. Species-level identification showed discrepancies between methods: all 80 isolates were identified as *E. meningoseptica *using the VITEK 2 Compact system, whereas MALDI-TOF MS identified them as *E. anophelis*. Demographically, the most commonly affected age group was 60-69 years, contributing 20.00% (16/80) of cases. Males were more frequently affected (61.25%, 49/80) than females (38.75%, 31/80). The isolates were evenly distributed between ICU and non-ICU settings, with 50% (40/80) from each (Table 1). The PCR results of all the archived isolates (80 *Elizabethkingia *spp.) are depicted in Table 2. All isolates produced a 281 bp PCR product, indicating the presence of *E. anophelis *(Figure 1). According to Chew et al., the expected sizes for *E. anophelis *and *E. meningoseptica *amplicons are 281 bp and 250 bp, respectively [6]. PCR results perfectly matched the MALDI-TOF MS identifications, showing 100% sensitivity and specificity in detecting *E. anophelis*.

**Table 1:** Summary of demographic, culture positivity, and identification results. NFGNB: non-fermenting Gram-negative bacilli, MALDI-TOF MS: matrix-assisted laser desorption/ionization time-of-flight mass spectrometry, ICU: intensive care unit.

Parameter	Findings
Total blood cultures processed	13,747
Culture-positive samples	1,869 /13,747 (13.59%)
NFGNB among culture positives	273/1,869 (14.60%)
*Elizabethkingia *spp. among NFGNB	80/273 (29.30%)
Identification by VITEK-2	*E. meningoseptica* (100%)
Identification by MALDI-TOF MS	*E. anophelis* (100%)
The most affected age group	60-69 years (20.00%)
Gender distribution	
Males	49 (61.25%)
Females	31 (38.75%)
ICU vs non-ICU source of isolates	
ICU	40 (50%)
Non-ICU	40 (50%)

**Table 2:** Results of species-specific PCR identification of Elizabethkingia. PCR: polymerase chain reaction.

Primers	Target	Amplicons	PCR result
anoR -5'-TGCGTTATTACCAGGTAGTCGG-3' anoF-5'-GACTTCCGCGGTAGCAAACAA-3'	E. anophelis	281 bp	Positive 100%
mengF - 5'-TGGGACCTATTGCTGTTGGTT-3' mengR (5'-ACCACTTCCTGTGTACCTGC-3')	E. meningoseptica	250 bp	Positive 0%

## Discussion

*Elizabethkingia *species are non-motile, non-fermenting, aerobic Gram-negative rods that cause various infections, including meningitis, bacteremia, pneumonia, and febrile episodes in neutropenic patients. These organisms exhibit unique antibiotic resistance patterns and sometimes respond to drugs typically used for Gram-positive bacteria [8].

Historically, *E. meningoseptica*, initially known as *Flavobacterium meningosepticum *and later *Chryseobacterium meningosepticum*, was a predominant pathogen, particularly in neonatal sepsis and meningitis among preterm infants [10]. Though mortality has declined over time, long-term neurological sequelae remain a concern [11].

However, it is now understood that many infections formerly attributed to *E. meningoseptica *were actually due to *E. anophelis*. This species has been implicated in various clinical conditions, including pneumonia, bacteremia, urinary tract infections, and catheter-associated infections. While it is mainly nosocomial, reports such as the Wisconsin outbreak suggest potential community acquisition [12].

Chang et al. found that the VITEK 2 system correctly identified *Elizabethkingia *species in 84.6% of cases, using 16S rDNA sequencing as a benchmark [13]. In another study, Chew et al. examined 79 isolates initially labeled as *E. meningoseptica *or *E. miricola *by MALDI-TOF and discovered via 16S rRNA sequencing that nearly all (98.7%) were actually *E. anophelis *[6]. This supports our finding of *E. anophelis* being more prevalent than previously assumed.

A Korean study further assessed species-level identification using two MALDI-TOF platforms and the VITEK 2 Gram Negative (GN) card, comparing them against 16S rRNA sequencing. Among 86 isolates, 59.3% were *E. anophelis*, while only the upgraded MALDI-TOF VITEK MS could accurately identify all strains. The VITEK 2 system showed limitations, correctly identifying only *E. meningoseptica *isolates [14].

Although sophisticated platforms such as MALDI-TOF MS, next-generation sequencing (NGS), and metagenomic sequencing provide fast and precise results, their widespread use is limited due to cost and accessibility, particularly in resource-constrained healthcare settings [15]. Japanese researchers have demonstrated that MALDI-TOF MS can rapidly differentiate *Elizabethkingia *species by detecting specific protein biomarkers such as L29, L30, S21, and YtxH, offering an edge over 16S rRNA-based methods [16]. Comparatively, *E. meningoseptica *often presents with higher minimum inhibitory concentrations (MICs) for several antibiotics [7]. In this study, antibiotic susceptibility differences between the two species of *Elizabethkingia *were not feasible to assess because the same isolates, previously identified as *E. meningoseptica*, were misidentified and found to be *E. anophelis*.

Given the high mortality rates (24-60%) associated with *E. anophelis*, accurate species identification is crucial for treatment decisions. Studies have shown that *E. anophelis* is more resistant and poses greater risks, especially in adults with comorbidities, whereas pediatric outcomes tend to be better [17-21].

To address diagnostic challenges, future investigations should focus on refining affordable and effective molecular diagnostics. Age-specific therapeutic approaches and continuous monitoring of antibiotic resistance patterns are essential for improving patient care and clinical guidelines. To the best of our knowledge, the existing knowledge regarding correct species identification of *Elizabethkingia *was based only on MALDI-TOF MS. This is the first study from India to perform species identification of *Elizabethkingia *using conventional PCR, and its performance was also evaluated against MALDI-TOF MS, thus highlighting the need for further research and standardization.

Limitations of this study are that it was a purely laboratory-based study conducted on archived isolates. Moreover, due to time constraints, the study was limited to 80 bloodstream isolates only. Future studies with larger sample sizes would be helpful to better understand its efficacy.

## Conclusions

Although MALDI-TOF MS remains the preferred method for identifying *Elizabethkingia *species, PCR-based detection offers a practical and reliable alternative in low-resource environments. Further research into enhanced molecular and sequencing techniques is essential for accurate diagnosis, ultimately contributing to better patient management and improved treatment outcomes.
